# Data by data, Big Data

**DOI:** 10.3325/cmj.2019.60.290

**Published:** 2019-06

**Authors:** Branimir K. Hackenberger

**Affiliations:** Department of Biology, Josip Juraj Strossmayer University, Osijek, Croatia *hackenberger@biologija.unios.hr*

The twentieth century was characterized by the development of the electronics industry and technology, and the new paradigms of contemporary science, the relativity theory and the theory of chaos. The beginning of 21st century has been marked with a rise of artificial intelligence (AI). The extreme investment in AI development in almost all developed countries is the latest news. AI became the new space race. In China, the city of Beijing only will allocate $ 2 billion to build its AI park. Investing in AI is an increasingly common theme in the Westminster Parliament, Duma, Bundestag, Diet, Congress, and other parliaments around the world.

Since 2010, AI-related industry has grown at a compounded growth rate of 60%. Simultaneously, the definition of AI is constantly developing. The simplest one is that AI is intelligence demonstrated by computers or any other artifact made by human or another machine in contrast to natural intelligence inherent in all kind of organisms. Computer, ie, machine, intelligence is based on neural networks, machine and deep learning. However, the success of each AI system depends primarily on the quality and amount of data used for its construction. Therefore, one of the essential components of AI is data processing technology. That is, the technique of processing the vast amounts of data commonly called Big Data. The term “Big Data” is relatively young. It has been used since the 1990s for the amount of data that is impossible to process with standard software tools. Because of a very fast developing of information technology, the definition of Big Data are permanently growing. Nowadays, the term Big Data are in use for any large amount of data that requires the use of specialized hardware and software tools, including the necessity of parallel computing. On the other hand, the term Big Data should be understood relatively. For example, if one wants to transfer 100 TB file using e-mail, this file has to be considered Big Data because nowadays no e-mail service enables a transfer of such a huge amount of data.

Big Data are becoming extremely important for their use in many branches connected to medicine, from genetic analysis, clinical laboratory practice to advanced medical analysis and the increasing quality of health care. The importance of Big Data has grown quickly because of more frequent usage of so-called streaming data. Streaming data are data generated from different sources, mostly sensors and various instruments, on an ongoing basis. Such data should be processed gradually through a stream processing techniques without the access to all data.

Monitoring devices used in intensive care units are a good example of the significance of streaming data technology. In the past, these readings have been summarized as one reading every few minutes or half an hour, but devices generate thousands of readings each second. Although these devices monitor very large amounts of data, much of that data was not available for analysis due to technology limitations. Using big data, eg, stream technology, the medical team can capture and process the data flow from the bedside monitors using algorithms designed to seek early warning signs of any significant physiological changes in patient’s organism. Real-time data can early warn about changes in a patient's condition, which can make it possible for doctors to help the patient as much as 24-72 hours earlier than would be possible without the data streaming technology.

According to Moore's law, the number of transistors in the most advanced integrated circuit is doubled nearly every two years. This is why more and more powerful computers are available. The internet's power and data power is growing. Clouds have become a common location for data storage. Even in less developed countries, almost all computer owners are connected to the internet. If we take into account that every internet user can generate data, we can imagine the amount of data that is being generated daily.

**Figure 1:**
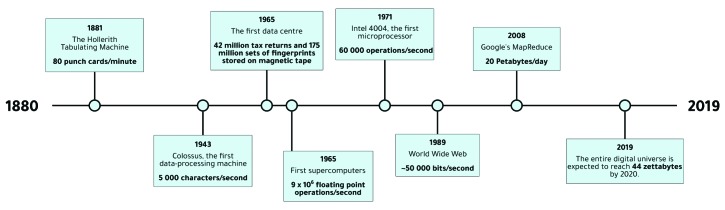
The rise of data toward Big Data.

The world is currently creating as many data in two days as humankind has created in over 2000 years. It is estimated that more than 2.5 trillion bytes of data per day are created, which means that in the last two years, up to 90 percent of all global “data” has been generated. (Figure 1) (Although, many data are distributed to the internet, many data remain on desktop computers or within local computer networks.)

The full power of Big Data technology is entirely apparent in well-known networking and data services offered by companies such as Amazon, Apple, Facebook and, of course, Google in the West and Alibaba, Baidu, and Tencent in China. The interesting fact is that most of these companies, as the basis for Big Data technology, use the same open-source service – Apache Hadoop. Apache Hadoop, in the most straightforward way, is a collection of open-source software utilities that make it easier to use a multi-computer network to solve problems involving massive amounts of data and computing. One of the most important features of big data technology is the existence of a large number of free or inexpensive basic software. More than ever, the programmer is expected to be very versatile and very knowledgeable. The processing of certain types of data requires not only the enthusiasm of programmers but also the detailed knowledge of the matter. Medical databases and processing are such examples. Besides Hadoop, there are two additional terms crucial for big data technology: MapReduce and NoSQL.

**Figure 2:**
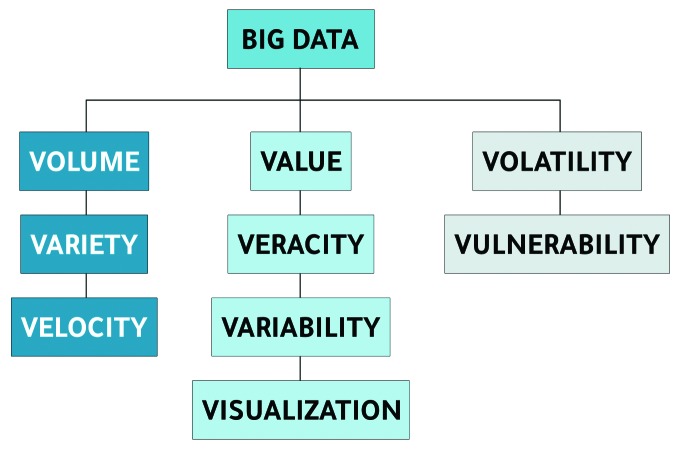
Nine Vs of Big Data.

Simply put, Hadoop is an open-source storage and a data storage framework for large data sets, which stores data via a so-called distributed Hadoop file system, while MapReduce takes care of the processing. MapReduce is, in other words, a software model that enables the processing of massive data stored in Hadoop. NoSQL (“no only SQL”) is a database design approach capable of accommodating a wide range of data models, including key value, document, columnar, and graph formats. NoSQL is an alternative to traditional relational databases in which data are placed in tables and data schemes are carefully designed before the database is built. NoSQL databases are particularly useful for working with large sets of distributed data. There is not just one but several measures that characterize Big Data. When Big Data technology started to develop, there were only three measures: volume, velocity, and variety. Over time, three more measures were added: visualization, veracity, and value. Lately, additional three measures – variability, volatility, and vulnerability, are often mentioned as characteristics of Big Data. So that today we can say Big Data are characterized by 9 Vs (1) (Figure 2).

*Volume* is the amount of data we have. Nowadays the size of Big Data are measured in zettabytes (10^21 bytes) or even in yottabytes (10^24 bytes). Modern equipment, sensors, and instruments, especially the Internet of Things (The Internet of Things, or IoT, is a system of interrelated computing devices, mechanical and digital machines, objects, animals, or individuals that have the ability to transfer data over a network without requiring human-to-human or computer-to-computer interaction.), create exponential data growth. *Velocity* is the velocity of access to data. *Variety* describes one of the biggest big data challenges as data can be unstructured and can include many different data types from small texts to very big pictures or videos. *Variability* of the Big Data refers to the meaning of the data, ie, the state of the data meaning. For example, if the combination of individual physiological parameters does not always have the same clinical significance, we say that Big Data with this data information is highly variable. *Veracity* is all about securing data accuracy, which requires processes to avoid the accumulation of bad data in systems. Nowadays, *visualization* is very important. Using charts and graphs to visualize massive amounts of advanced knowledge in a conveyance is far more practical than spreadsheets and reports stuffed with pure numbers. The *value* is the usefulness (the cost) of results that the data will give after processing. *Volatility* is how old data need to be before they are considered irrelevant or not useful any longer. *Vulnerability* refers to the security of information stored in databases that will be used for Big Data.

Volume, velocity, and variety are not only the key parameters of Big Data, but also the key features of distinction between “normal” data and Big Data. Variability, veracity, visualization, and value are important attributes that reflect Big Data's complexity for those who would process, analyze, and benefit from it.

Big Data studies mostly seek hidden patterns and information in a large amount of complex data. There are four types of Big Data studies: enriched, combined, and augmented studies and virtual interventions. In enriched surveys the original data are being strengthened with additional data often from open access databases. Combined studies, on the other hand, merge personal data, often obtained from medical devices or remote sensors with other data connected with the task of interest. In augmented studies, data from database studies are reused or analyzed with data analysis methods used in AI technology. Through virtual interventions, data collected from clinical trials and interventions are reused for simulation of possible real-life scenarios (2).

Big Data represents a new perspective in medical research due to the necessity of large amount of data and interdisciplinary approach. Moreover, Big Data enables fast extraction of information relevant for specific task from vast quantities of heterogeneous, unstructured, and distributed data. As a consequence of its characteristics and potentials, Big Data in medicine should be based on both personalized, multi-scale approaches and social approaches that are based on observations and aimed at predicting and explaining events (3).

R, as the informal standard for statistical processing of biomedical data, has also the ability for Big Data analysis. The pbdDMAT R package's main objective is to provide R users with an access to highly strong distributed, implicitly parallel computing, while maintaining the friendly and familiar R syntax for these computations, so that many current R codes can be used with this scheme with only trivial changes, yet receive massive performance boosts (4). Furthermore, RHIPE is an R package (5) that provides a way to use Hadoop from R. Today a very popular programming language is Python. Although the statistical power of Python lags behind R, Python is a very powerful Big Data tool. Python package Optimus enables preparation, exploration, and creation of machine learning models for Big Data, but there are more other libraries that offer solutions for Big Data such are Pandas, Agate, and Bokeh (6) (7). Big Data are future that began yesterday. Today, thanks to the amount of free software, it is possible to work with Big Data technology on even a better personal computer.
